# Abernethy at the Last

**Published:** 1840-06

**Authors:** 


*Abernethy at the last.*—His eccentricity continued during his existence, and towards the last he is reported to have joked upon the œdematous state of his legs produced by the disturbance of the circulation and his difficulty of breathing. Some one inquired of him how he was? to which he replied, " Why, I am better on my legs than ever: you see how much stouter they are?" His hobby retained full possession also to the end of his life. He attributed his disease to the stomach. He said, " it is all stomach; we use our stomach ill when we are young, and it uses us ill when we are old." But it is not a little singular, that he expressly enjoined that no examination of his body should take place !—*Medico-Chirurgical Review.*

